# Efficient Amino Acid Conformer Search with Bayesian Optimization

**DOI:** 10.1021/acs.jctc.0c00648

**Published:** 2021-02-12

**Authors:** Lincan Fang, Esko Makkonen, Milica Todorović, Patrick Rinke, Xi Chen

**Affiliations:** Department of Applied Physics, Aalto University, AALTO 00076, Finland

## Abstract

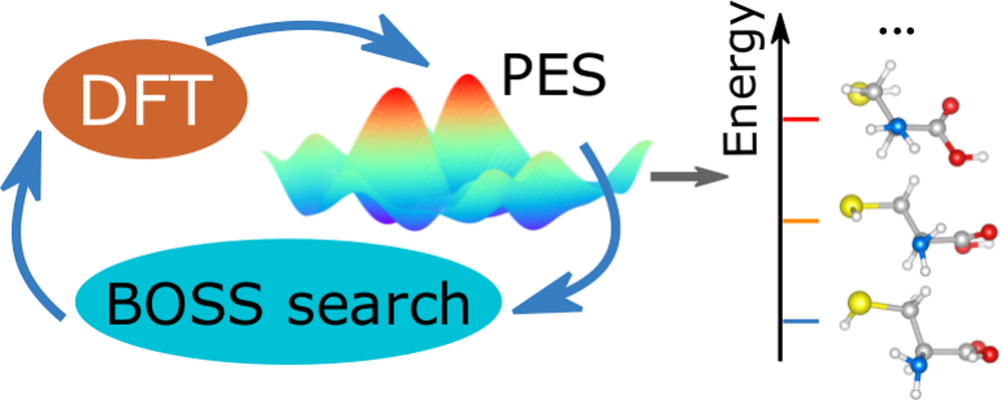

Finding low-energy molecular conformers is challenging due to the high dimensionality of the search space and the computational cost of accurate quantum chemical methods for determining conformer structures and energies. Here, we combine active-learning Bayesian optimization (BO) algorithms with quantum chemistry methods to address this challenge. Using cysteine as an example, we show that our procedure is both efficient and accurate. After only 1000 single-point calculations and approximately 80 structure relaxations, which is less than 10% computational cost of the current fastest method, we have found the low-energy conformers in good agreement with experimental measurements and reference calculations. To test the transferability of our method, we also repeated the conformer search of serine, tryptophan, and aspartic acid. The results agree well with previous conformer search studies.

## Introduction

A molecular conformer is a distinct conformation corresponding to a minimum on the molecule’s potential energy surface (PES). Any molecule with rotatable bonds has several stable conformer structures, each associated with different chemical and electronic properties. At ambient temperatures, all the properties of that molecule are the combination of the properties of its conformers accessible at the temperature of the study.^1−3^ Therefore, identifying the low-energy conformers and determining their energy ranking continues to be a topic of great interest in computational chemistry,^4^ cheminformatics,^5,6^ computational drug design,^7^ and structure-based virtual screening.^8^ While one configuration of a small molecule can be simulated routinely by *ab initio* methods, the large size of configurational phase space and the considerable number of local minima in typical energy landscapes make conformer searches one of the persistent challenges in molecular modeling.^1,5,6^

The first challenge in conformer search is sufficient sampling of the configurational space. The conformational space (bond lengths, bond angles, and torsions) for even relatively small molecules is enormous.^9,10^ For this reason, dimensionality reduction is commonly applied to make the problem more tractable. Since the bond lengths and angles are relatively rigid in molecules and the different conformers originate from the flexible rotational groups, most search methods focus on sampling the torsion angles in molecules while keeping bond length and angles fixed.^1^ A variety of methods and tools have been developed to generate diverse conformer structures.^11−16^ These methods can be broadly classified to be either systematic or stochastic.

A systematic method relies on a grid to sample all the possible torsion angles in the molecule. This approach is deterministic but limited to small molecules because it scales poorly with increasing numbers of relevant torsion angles, i.e., search dimensions. Stochastic methods randomly sample the phase space of torsion angles (sometimes restricted to predefined, most relevant ranges) based on different algorithms such as Monte Carlo annealing,^17,18^ minima hopping,^19^ basin hopping,^20,21^ distance geometry,^22^ and genetic algorithms.^11,23^ Stochastic methods can be applied to larger molecules with high-dimensional conformer spaces, but the predicted conformers may vary. Extensive sampling is required, and the results may be affected by the random nature of the process.

Knowledge-based methods have also been developed^24,25^ to achieve more consistent results. They use a predefined library for torsion angles and ring conformations. The library is typically based on experimental structures in databases such as the Cambridge Structure Database (CSD)^26^ or the Protein Data Bank (PDB).^27^ To search the conformers, knowledge-based methods usually need to be combined with the different systematic or stochastic algorithms mentioned before.

The second challenge in conformer searches is the sufficiently accurate mapping of energies and structures. Two classes of total energy approaches are commonly used: force field-based methods and quantum chemistry methods such as the density functional theory (DFT) and coupled cluster (CC) theory. Quantum chemistry methods achieve higher accuracy in the estimation of molecular energies than force fields because they describe the interactions and polarization in molecules more accurately. However, they are computationally costly. More often than not, quantum chemistry methods are too expensive to provide energies for all configurations produced in the search.

To balance efficiency and accuracy, hierarchical methods have been developed. Fast computational methods with lower accuracy are employed to to scan the configurational space. Promising candidate structures are then funneled through more costly methods with higher accuracy to refine the conformer structures and energies (such as force fields → DFT^28,29^ or HF → MP2 → CCSD(T)^30^). Methods at different levels predict different PESs. To avoid missing the true low-energy conformers, a large portion of configurational space has to be sampled at a lower accuracy method level, and many structures need to be optimized at a higher level.

In recent years, artificial intelligence (AI) and machine learning (ML) techniques such as genetic algorithms,^31,32^ artificial neural network,^33,34^ Gaussian process regression (GPR),^35−37^ and machine-learned force fields^38^ were used to accelerate structure-to-energy predictions and geometry optimization for molecules. The majority of these schemes requires a large number of data points, which may be costly to compute with *ab initio* methods. To reduce the amount of required data, Bayesian optimization was introduced in the structure search.^39−41^ Bayesian optimization search schemes belong to the active learning family of methods, which generate data on the fly for optimal knowledge gain.

In this article, we present a new procedure for molecular conformer identification and ranking. We combined the Bayesian optimization structure search (BOSS) approach^40^ and quantum chemistry simulations to find the conformers of small molecules and accurately predict their relative stability. BOSS is a python-based tool for global phase space exploration based on Bayesian optimization.^42^ Beyond the Bayesian active learning method for the global minimum conformer search in ref (39), our procedure aims to find all the relevant conformers in one run. We use cysteine as a model system to demonstrate our methodology and then later generalize to other amino acids.

**Figure 1:**
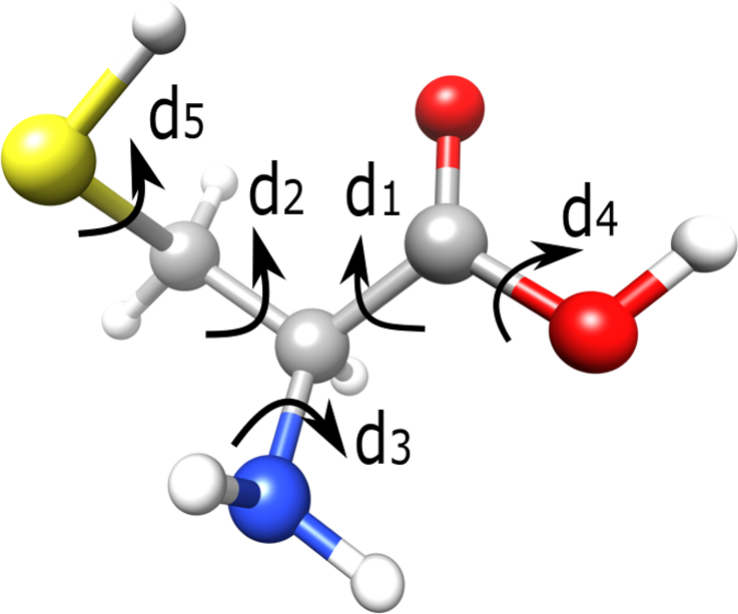
Ball-and-stick model of the cysteine molecule. Red is used for oxygen, white for hydrogen, gray for carbon, blue for nitrogen, and yellow for sulfur. *d*_1_, *d*_2_, *d*_3_, *d*_4_, and *d*_5_ label the five dihedral angles of cysteine that we use to define our search space.

Cysteine was chosen for several reasons. First, it is an amino acid with critical biological functions. Second, it is the only amino acid that has a −SH group. The strong S–Ag and S–Au bonds make it interesting for use in hybrid nanomaterials.^43,44^ Third, cysteine has five rotational groups, as shown in Figure 1. Therefore it is an interesting and accessible five-dimensional (5D) system for Bayesian optimization. Last, the structures and the energy order of cysteine’s conformers have been calculated by several groups using the grid sample method^30,45,46^ and characterized by Fourier transform microwave spectroscopy experiments^47^ so that we can compare the accuracy and efficiency of our new procedure with other computational and experimental results.

In brief, using cysteine as an example, we present an efficient and reliable procedure to predict the structures and energies of molecular conformers. BOSS ensures sufficient sampling of the configurational phase space and outputs the structures associated with local energy minima. We post-processed the machine-learned conformer candidates with geometry optimization and then added free energy corrections to obtain the final ranking. We tested the effect of different exchange-correlation functionals and van de Waals interactions on the ranking order. Finally, we applied coupled cluster corrections to the lowest-energy conformers.

To test the generalizability and transferability of our method, we also studied the conformers of three other amino acids: tryptophan, serine, and aspartic acid. Serine and tryptophan have a five-dimensional phase space for our purposes, and aspartic acid has 6 rotational degrees of freedom. The methods and results will be presented in the following sections.

## Methods

### BOSS-based Molecular Conformer Search

**Figure 2:**
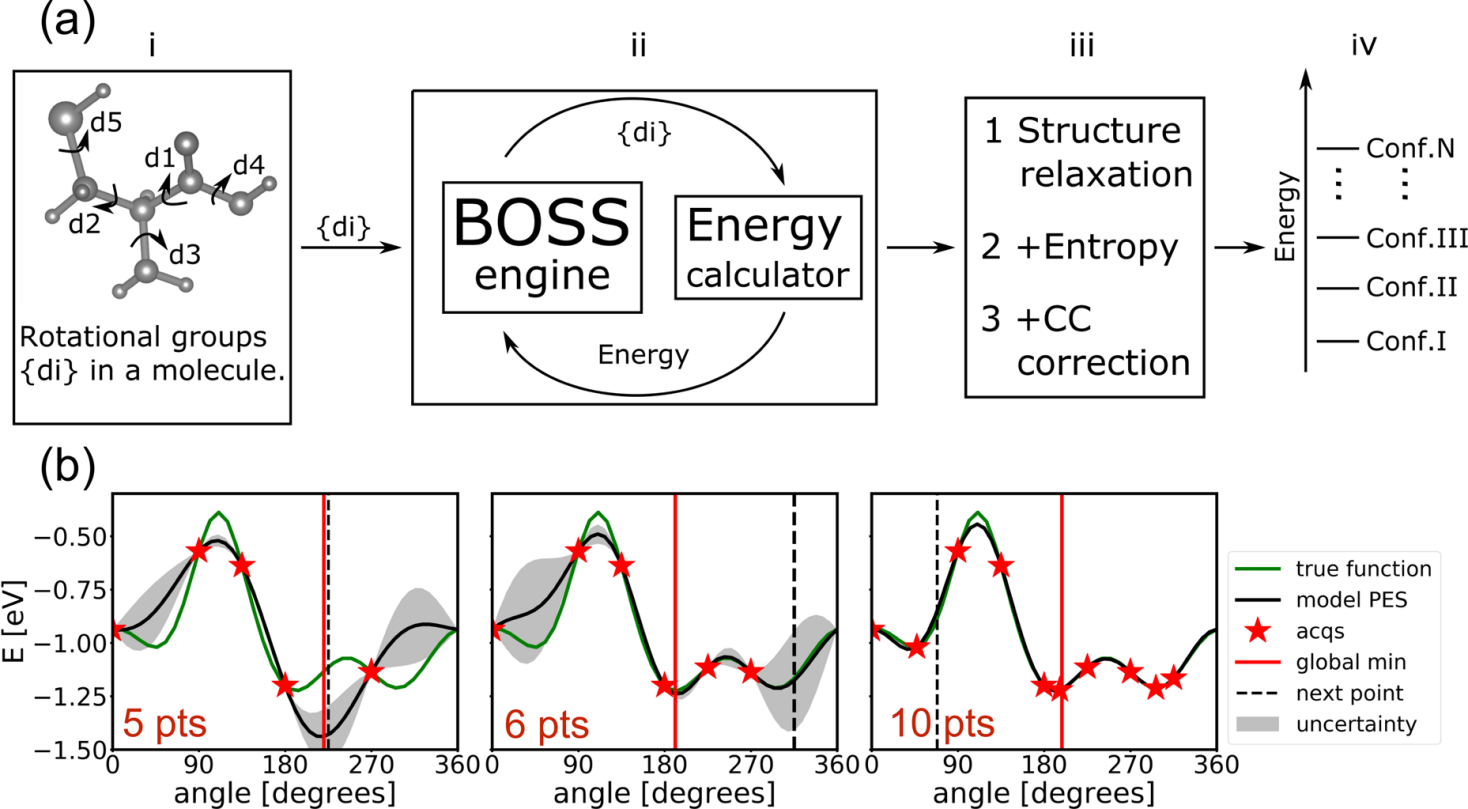
(a) Overview of our BOSS-based procedure for molecular conformer search, featuring (i) system preparation, (ii) Bayesian optimization conformer search, (iii) refinement, and (iv) validation. (b) BOSS iterative inference of a one-dimensional (1D) PES of the *d*_1_ dihedral angle of cysteine. The GP’s native uncertainty (gray areas) facilitates exploratory data sampling. The global minimum location and the entire PES are learned in 10 data acquisitions.

Our BOSS-based procedure for molecular conformer search contains four steps: (i) System preparation, (ii) Bayesian optimization conformer search, (iii) refinement, and (iv) validation, as illustrated in Figure 2a.

In step (i), we first obtain an xyz-file of our molecule of interest from a database and then perform a single geometry optimization with a quantum chemistry method. Then, we calculate the *z*-matrix to find the dihedral angles. We chose the dihedral angles d*_n_* to describe the different conformers, as they are typically the most informative degrees of freedom for conformers. We keep all bond lengths and angles fixed at their optimized values. Such dimensionality reduction is standard practice to expedite the molecular conformer search, as mentioned in the Introduction.

In step (ii), BOSS actively learns the PES of the molecule by Bayesian optimization iterative data sampling. Each data “point” consists of the set of dihedral angles d*_n_* for a molecular configuration and its corresponding total energy *E*. In this step, we use DFT as the calculator. *E* therefore corresponds to the DFT total energy of a molecular configuration.

BOSS employs Gaussian process (GP) models^48^ to fit a surrogate PES to the data points, and then refines it by acquiring more data points at locations that minimize the exploratory lower confidence bound (eLCB) acquisition function.^42^ The most-likely PES model for the given data is the GP posterior mean. The lack of confidence in the model is reflected by the GP posterior variance, which vanishes at the data points and rises in unexplored areas of phase space. The key concepts of this active learning approach are illustrated in Figure 2b, in which BOSS iteratively infers a one-dimensional PES of the *d*_1_ dihedral angle of cysteine. The global minimum location and the entire PES are learned in 10 data acquisitions. In analogy with the 1D example, BOSS actively learns the PES in *N* dimensions until convergence is achieved. The advantage of BOSS is not only its efficiency but also the fact that it explores both the global minimum and local minima of the PES during the search. We exploit this feature to find conformers beyond the global minimum, which we associate with the local minima of the PES. A more detailed introduction of the BOSS approach can be found in refs (40, 49), and.^50^

The current BOSS implementation does not restrict the search space, which for rotational degrees of freedom may result in steric clashes. For example, for aspartic acid and tryptophan, BOSS occasionally samples physically non-meaningful structures with very high energies. Such energy spikes can obstruct model fitting and should be avoided. In this work, we refrain from restricting the search space and instead apply an energy transformation: *E*_new_ = *E*_cut_ + log_10_(*E*). If the DFT energy *E* of a given structure is higher than *E*_cut_, we damp it down by taking the logarithm. We tested *E*_cut_ = 1.0, 2.0, 3.0, and 4.0 eV for aspartic acid and found 2.0 eV to be optimal. BOSS hyper-parameters converge fastest for this *E*_cut_ value, and only 0.017% acquisitions needed to be transformed (Figure S7). We therefore adopted *E*_cut_ = 2 also for the other amino acids.

After the BOSS-predicted PES has converged, in step (iii), we analyzed the PES to extract the local minima locations and related structures. Since the PES and its gradients can be computed efficiently at any location in the *N*-dimensional phase space from the GP model, BOSS post-processing routines perform multiple L-BFGS (limited-memory Broyden–Fletcher–Goldfarb–Shanno algorithm) minimizations, using the locations of the data acquisitions as starting points. Because models built with more datapoints tend to be more complex and feature more minima, starting numerous minimisers from different points allows us to identify as many different minimum basins as possible in the PES surrogate model. This procedure potentially reports the same minima multiple times. For this reason, we developed automated duplicate purging routines to output only different minima after postprocessing (typically about 10% of all minima found). The resulting shortlist of minima may still contain similar structures, and the final pruning is left to the user, as required by the application.

Next, we refine the local minima output by BOSS by geometry optimization and entropy corrections. First, all degrees of freedom (including bond lengths and angles) are relaxed to obtain optimized structures and energies. Next, we add vibrational entropy corrections following previous studies.^51,52^ We compute and add the zero-point energy and the vibrational free energy at 300 K to the energies of optimized conformers. Since most experiments are performed at room temperature, we picked a temperature of 300 K for the vibrational corrections.

In step (iii), we also go beyond DFT. We perform coupled cluster calculations for the DFT-optimized conformer structures in a relevant energy window. Coupled cluster (CC) theory is an approximate infinite-order perturbation theory, in the form of exponential cluster operators describing the quantum many-body effects of the electronic wave function. Despite being significantly more expensive than DFT and scaling polynomially with system size, CC theory provides a systematically improvable hierarchy of approximations for accurate energy predictions. Due to the high computational cost, we only apply the CC method to the low-energy conformers we are interested in. The difference between the coupled cluster and DFT total energy, here called CC correction, is then added to the entropy corrections we added earlier in step (iii).

In step (iv), we validate our results by comparing the low-energy conformers we found to experimental and other computational results. System preparation and final validation require human input, but procedures featuring structure search and refinement can be made fully automated into a computational workflow.

### Computational Methods

In this work, we employed DFT as the predominant energy calculator and employed the all-electron code FHI-aims^53−55^ for all DFT calculations. ″Tight″ numerical settings and ″tier 2″ basis sets were used throughout. To investigate the influence of the exchange-correlation functional and the level of dispersion correction on the final results, we performed our conformer search with the PBE + TS,^56,57^ PBE + MBD,^56,58^ PBE0 + TS,^57,59^ and the PBE0 + MBD^58,59^ functionals. For geometry optimizations, the geometry was considered to be converged when the maximum residual force (fmax) was below 0.01 eV/Å. To ensure that this fmax setting is tight enough, we have performed test calculations with fmax = 0.0001 eV/Å. The root mean square (RMS) difference of all atomic coordinates is 0.00036 Å, and the energy difference is 0.000003 eV.

Vibrational free energies were computed using the finite-difference method within the harmonic approximation. We used a finite-difference displacement length of δ = 0.0025 Å. The vibrational free energy *F*_vib_ was then calculated as follows
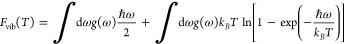
1where *g*(ω) is the phonon density of states and *T*, ω and *k_B_* are the temperature, frequency, and Boltzmann constant, respectively.

Going beyond DFT, we performed CC calculations with single, double, and perturbative triple excitations (CCSD(T)). These were done as single-point calculations using the structures from the PBE0 + MBD calculation with aug-cc-pVTZ basis sets. For validation purposes, we also performed MP4 and MP2 single-point calculations for selected conformers in their PBE0 + MBD geometries with 6-311++G(d,p), 6-311++G**, or aug-cc-pVTZ basis sets. We used the Gaussian16 code^60^ for the CCSD(T), MP4, and MP2 simulations.

To support open data-driven chemistry and materials science,^61^ we uploaded all calculations of this work to the Novel Materials Discovery (NOMAD) laboratory.^62^

### 2D Test

**Figure 3:**
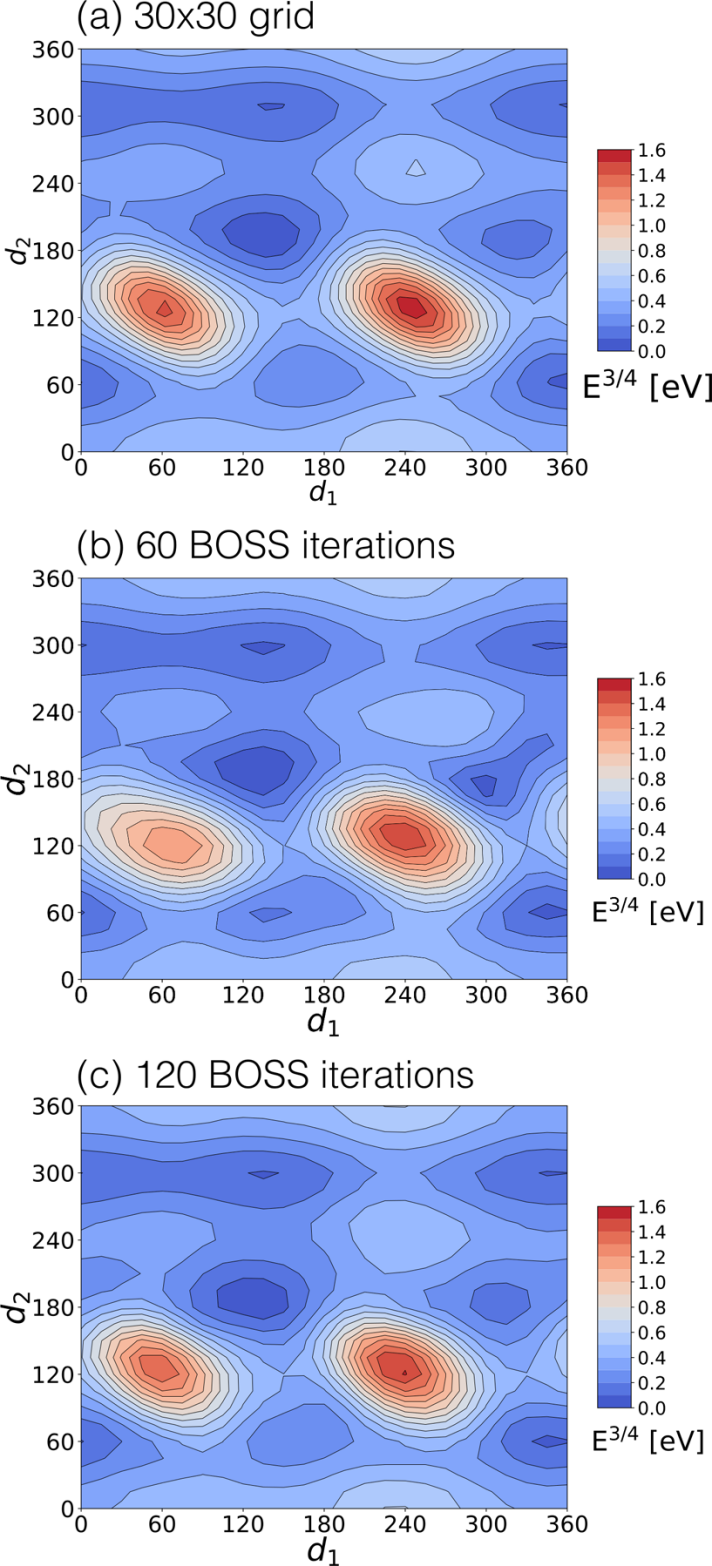
2D (*d*_1_, *d*_2_) PES map of cysteine generated by (a) 30 × 30 = 900 DFT single-point energy calculations, (b) 60 BOSS iterations, and (c) 120 BOSS iterations. To increase the PES contrast, *E*^3/4^ instead of *E* is plotted.

To test the accuracy and efficiency of step (ii) in our procedure, we started with a 2D search case in cysteine (Figure 3). First, we rotated the *d*_1_ and *d*_2_ dihedral angles to generate a reference map on a fine grid (30 × 30 points, Figure 3a). Then, *d*_1_ and *d*_2_ were sampled by BOSS. In both approaches, the bond lengths, bond angles, and other dihedral angles (*d*_3_ =180.03, *d*_4_ = 145.59, *d*_5_ = 180.03) were fixed in their DFT-optimized values. We obtained the energy of each structure with single-point PBE0 + MBD calculations and then plot the energy relative to the global minimum.

The 2D PES maps after 60 and 120 data acquisitions are shown in Figure 3b,c. Looking at Figure 3, we find that BOSS captures correct minima and maxima already after 60 data acquisitions (6% of the computational cost of the grid method), while after 120 data acquisitions, the BOSS PES resembles the reference map very well. This 2D PES features 6 energy minima of similar depth, suggesting considerable complexity of cysteine conformational phase space and many competing minima. We apply abundant sampling in high-dimensional problems so that we can recover all relevant conformer solutions.

### Cysteine Conformer Search in 5D

After demonstrating the BOSS rationale in 1D and 2D, we proceed to five dimensions. The five dihedral angles (*d*_1_–*d*_5_) in cysteine were sampled simultaneously by BOSS, and the energies of the corresponding configurations were evaluated with the PBE0 + MBD functional.

**Figure 4:**
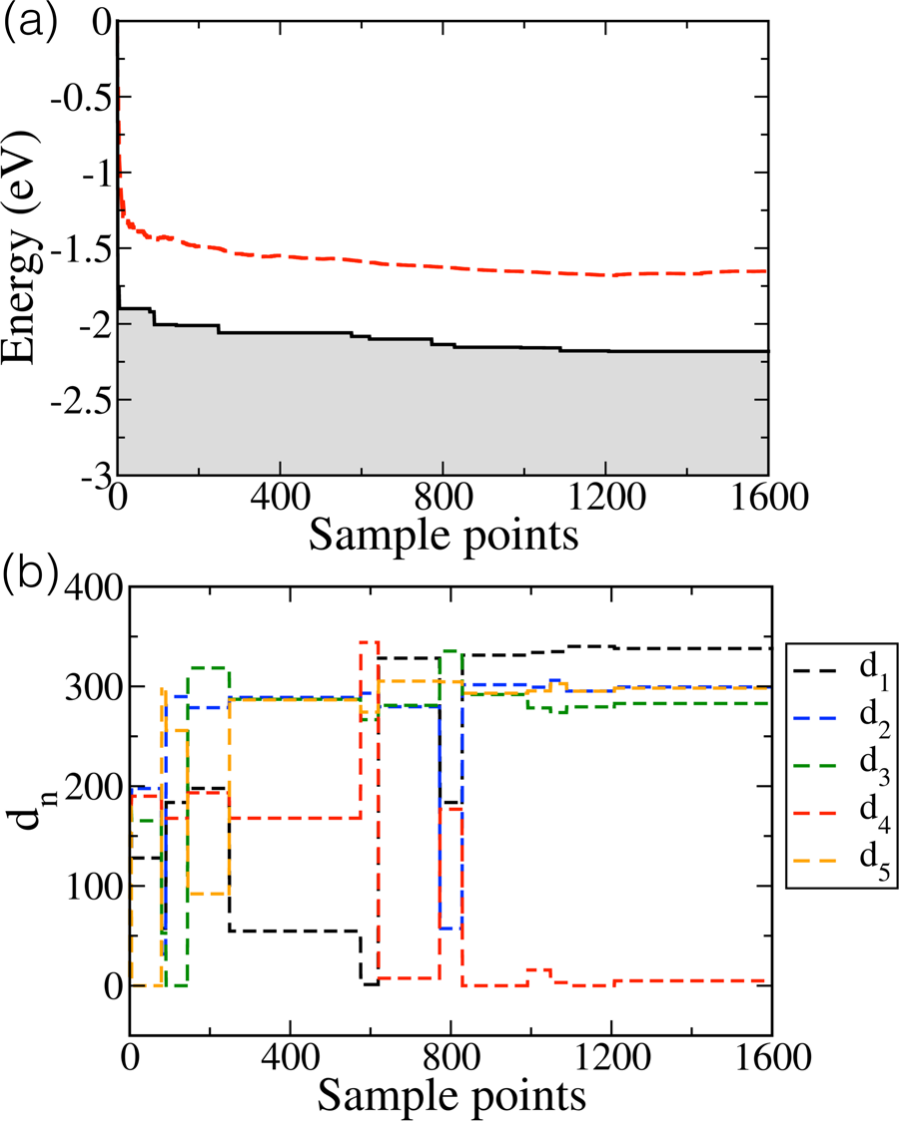
(a) Convergence of the global minimum energy computed from the BOSS-predicted global minimum configuration (black line). The average computed energy of the sampled conformers is shown with a red dashed line. (b) Value of the dihedral angles *d*_n_ of the BOSS-predicted global minimum as a function of the number of sampled points.

Figure 4 illustrates the refinement of the predicted global minimum with iterative configurational sampling. The lowest observed energy (calculated from the BOSS-predicted global minimum conformer) is shown in Figure 4a, and the values of the corresponding dihedral angles *d*_n_ is shown in Figure 4b. The lowest energy observed decreases continuously. Throughout the procedure, the geometry of the global minimum conformer changes, as Figure 4b illustrates. The global minimum undergoes several refinements until, at iteration 830, both the energy and the dihedral angles are converged and only have negligible changes (Δ*E*< 0.025 eV and Δ*d*< 10°).

Improvements of the global minimum prediction is due to instances of visiting low energy configurations chosen smartly form a vast 5D space. However, most model refinements proceeded with higher energy conformers and explores local minima of the PES, on average in the region 0.4 eV above the predicted global minimum, as shown by the red dashed line in Figure 4a.

Next, we address the convergence of the low energy part of the PES. This is not a trivial task, as we cannot monitor the PES in every point of the 5D space. It also turns out to be impractical to track the dihedral angles of several low energy conformers and monitor convergence as we did for the global minimum. The reason is that many conformers are very close in energy and switch order as the iterations progress. We therefore decided to take the energy-versus-conformer-number curve as the convergence indicator.

**Figure 5:**
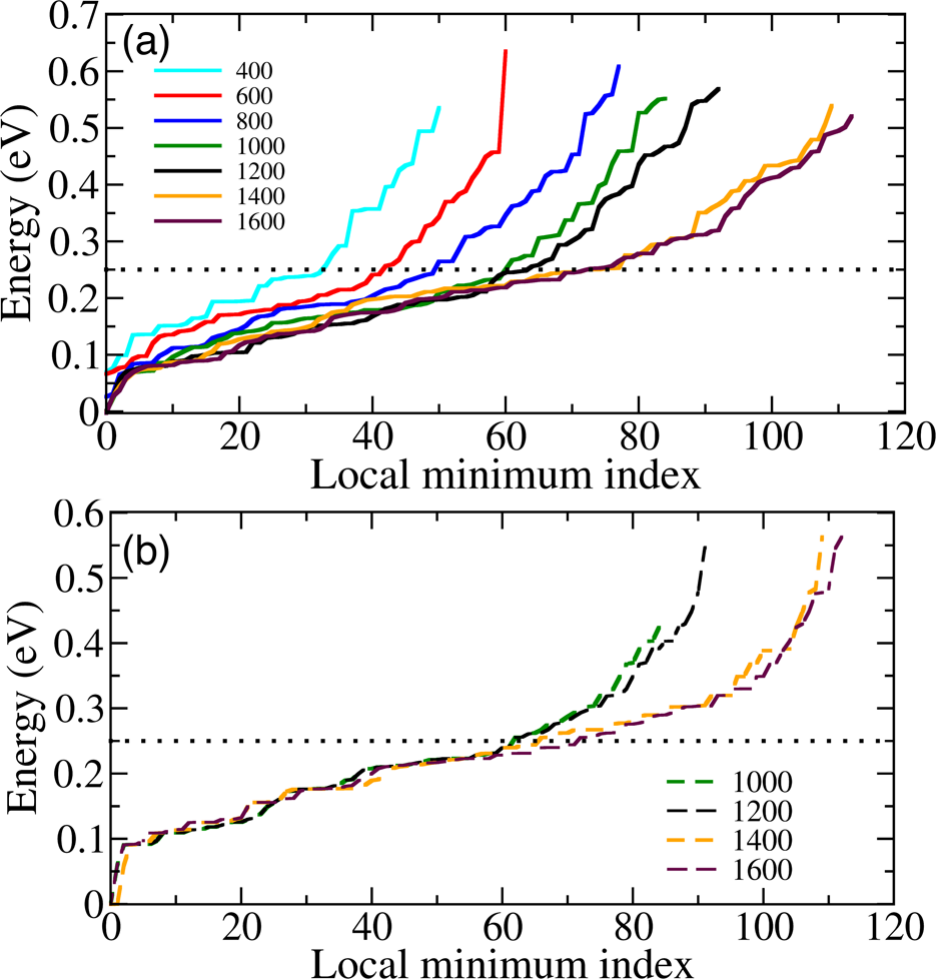
(a) Progression of the relative energy of predicted local minima for a PBE0 + MBD BOSS run with a total number of 1600 iterations. Shown are intermediate curves at 400, 600, 800, 1000, 1200, 1400, and 1600 iterations. (b)We took the conformers from 1000, 1200, 1400, and 1600 iterations and did the DFT structure optimization with PBE0 + MBD. The conformers are reordered from the lowest to the highest energy.

Figure 5a shows the relative energy of all local minima after 400, 600, 800, 1000, 1200, 1400, and 1600 BOSS iterations. BOSS uses the acquisition locations as starting points for local energy minimizations on the PES, so the number of minima found tends to increase as the iteration steps increase. In the figure, 0 eV is set to be the lowest energy found in the 1000th iteration. The curves after only 400 and 600 iterations still rise steeply and feature the wrong global minimum (i.e., do not start at 0 eV). With increasing number of iterations, the curves gradually approach the curve for 1200 iterations. At 1000 iterations, the curve is very similar to that of 1200 iterations in the low energy region (<0.25 eV), which suggests that not only the global but also the low-energy local minima conformers are converged. When the BOSS iterations increase to 1400 and 1600, more local minima were found in the higher energy region (>0.25 eV), but few changes are observed below 0.25 eV. Further proof of this is presented in the Supporting Information, where we show the 2D (*d*_1_, *d*_2_)-projected and (*d*_3_, *d*_4_)-projected BOSS-predicted PESs in Figures S1 and S2. The similarity of the 2D PESs at 1000 and 1200 iterations again suggests that the model is sufficiently converged in the low energy region at 1000 iterations.

We then extracted all conformers from runs up to 1000, 1200, 1400, and 1600 iterations and performed DFT geometry optimizations for all structures. The corresponding energy vs conformer index curves are shown in Figure 5b. Now the different lines lie almost on top of each other below 0.25 eV, confirming our PES in the low-energy region is sufficiently converged for 1000 iterations.

Next, we use the optimized structures at 1000 BOSS iterations and include the vibration energy as described in BOSS-based Molecular Conformer Search. Finally, we apply CCSD(T) single-point corrections to the 15 lowest energy conformers obtained from the PBE0 + MBD calculations.

## Results and Discussion

Using the methodology introduced in the previous sections, we performed four independent conformer searches with the PBE + TS, PBE + MBD, PBE0 + TS, and the PBE0 + MBD functionals for cysteine. In this section, we systemically assess how the different exchange-correlation functionals and van de Waals corrections affect the results and discuss how the different steps improve accuracy. We also compare our predictions with the experimental results and reference calculations.^30,47^

**Figure 6:**
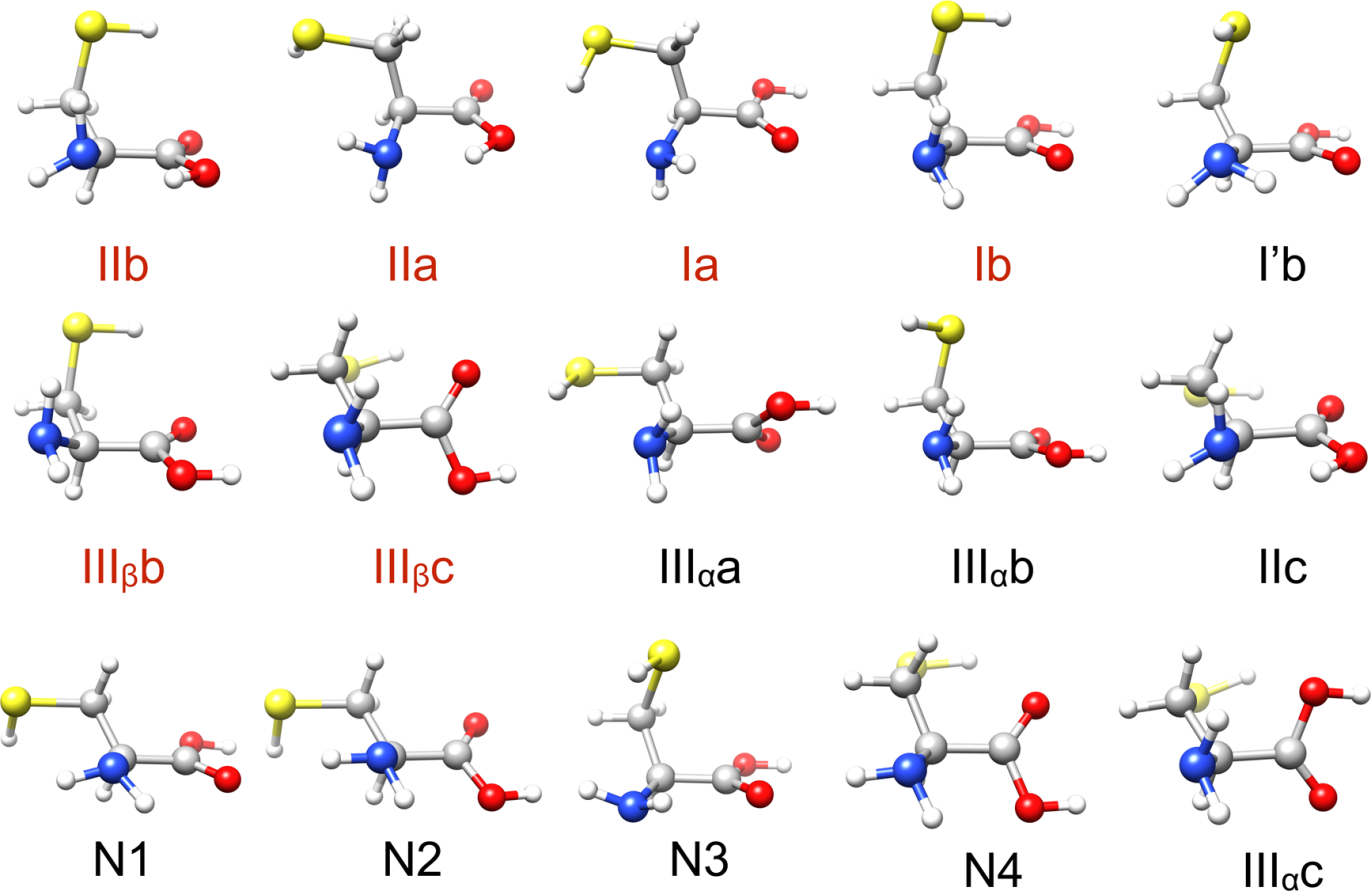
Predicted low energy conformers of cysteine from the PBE0 + MBD search. Conformers are named as I (NH–O=C), II (OH–N), and III (NH–OH) depending on the type of the hydrogen bonds, and as a, b, or c depending on the configuration of the −CH_2_SH side chain, following ref (47). The experimentally detected conformers are marked in red and other conformers marked in black. The colour scheme of the atoms is the same as in Figure 1.

We chose two references to make the comparison and validate our results. Reference^47^ reports both experimental and computational results. The computational energy ordering is obtained from single-point MP4 calculation on MP2 optimized structures using 6-311++G(d,p) basis sets. In the reference, six experimental conformers were found by rotational spectroscopy (labeled in red in Figure 6); five other low-energy conformers were predicted from the MP4 simulations but were not detected in the experiment (labeled in black in Figure 6). The authors of ref (30) did a systematic scan of 11,644 initial structures at the HF/3-21G level, located 71 unique conformers of cysteine using the MP2(FC)/cc-pVTZ method, and finally determined the relative energies of the 11 lowest-energy conformers with CCSD(T). Reference^30^ also provides *xyz*-coordinates for the observed conformers.

### Conformational Energy Hierarchy of Cysteine

The predicted 15 lowest energy conformer structures of cysteine with the PBE0 + MBD functional are shown in Figure 6. The atomic coordinates of the conformers can been found in the Supporting Information. To directly compare our results with those reported in ref (47), we assign our structures the same labels as ref (47). All the 11 conformers in ref (47) have been identified in our simulations within an energy window of 0.2 eV from the global minimum. In addition, BOSS predicted new conformers, which we named N1, N2, .... Some of them are shown in Figure 6. The new conformers BOSS predicted generally have a higher energy.

**Figure 7:**
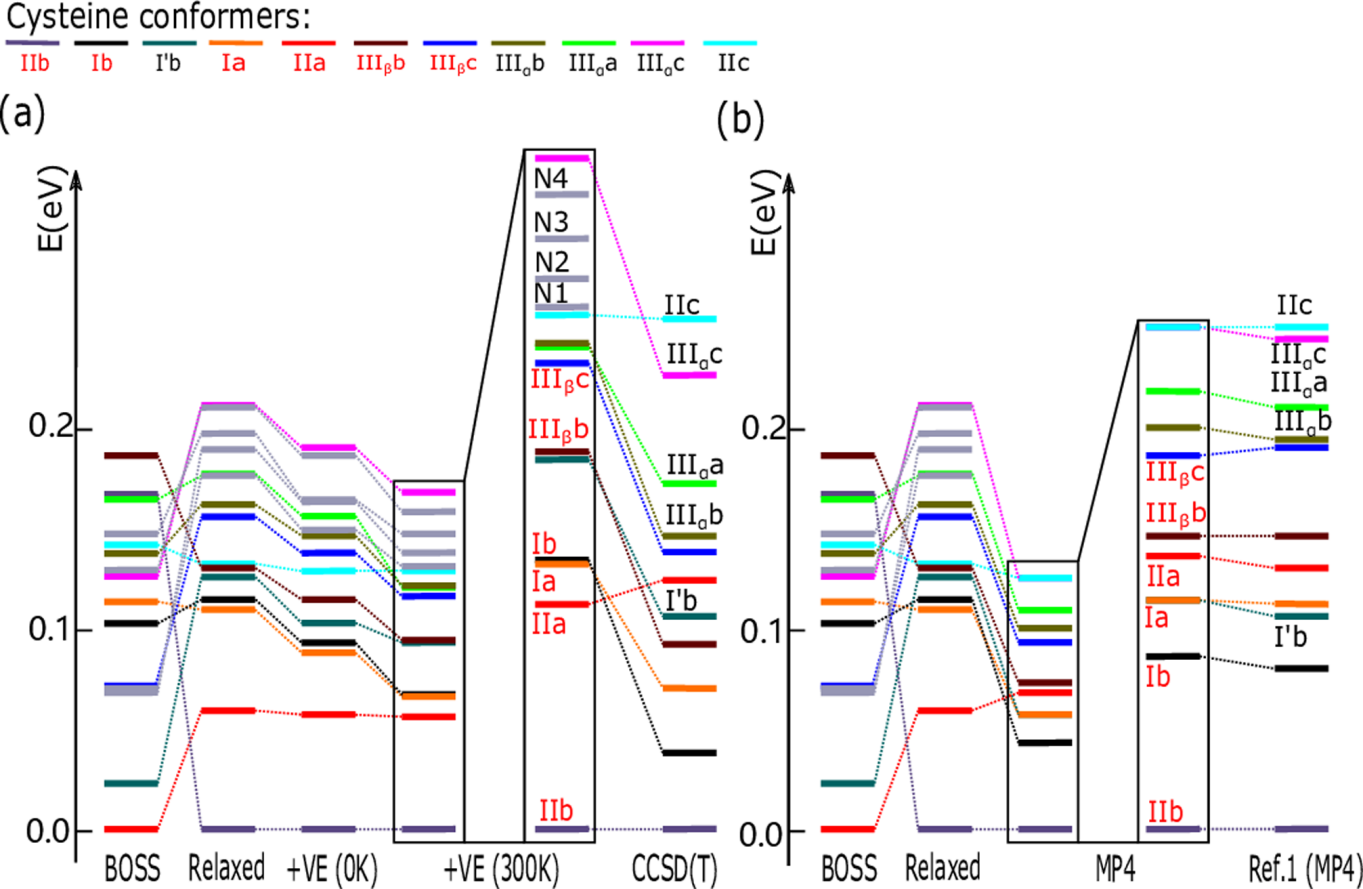
Relative stability for all steps of the PBE0 + MBD-based search. (a) From left to right: BOSS prediction, after structure optimization, after adding the vibrational energy at 0 K (+VE (0 K)), and adding the vibration energy at 300 K (+VE (300 K)). The two most right ones are +VE (300 K) and the energy order of CCSD(T) result but enlarged two times. For each step, the energy of the most stable structure defines the zero of energy for that column. (b) From the left to right: BOSS prediction, after optimization, and MP4 energy calculations. The last two columns show an enlarged version of the MP4 results in comparison with the MP4 results of Ref (47).

The relative stability of the PBE0 + MBD conformers is shown in Figure 7a. Corresponding plots for the PBE + TS, PBE + MBD and the PBE0 + TS functionals are presented in Figures S4–S6 of the Supporting Information. To illustrate the importance of different contributions to the energy hierarchy, Figure 7a and Figures S4–S6 show not only the final energy order but also intermediate steps.

The hierarchy figures show that once the conformers are extracted, geometry optimization plays a major role in refining their energy ranking. The largest energy changes and reordering happens in this step. This is expected because BOSS models rely on the fixed bond lengths and angles (building block approximation). In the PBE0 + MBD simulation, the average energy change of the most stable 15 conformers during the geometry optimization is 0.095 eV, while the dihedral angles of the corresponding structures change on average by Δ*d*_1_ = 16.9°, Δ*d*_2_ = 20.9°, Δ*d*_3_ = 8.9°, Δ*d*_4_ = 26.1°, and Δ*d*_5_ = 11.9°. How the geometry optimization changes the total energy of individual conformers can be seen in Figure S3.

The entropy corrections have a smaller effect on the conformer ordering. The zero-point energy contributions (+VE (0 K) column) does not trigger any conformer reordering. It does, however, compress the energy spectrum as corrections for higher-energy conformers are larger than for the global minimum. The finite temperature corrections (+VE (300 K) column) leads to a further compression of the energy spectrum. Now a couple of conformers above 0.1 eV switch orders as their vibrational entropy contributions differ.

The final column in Figure 7a shows our most accurate conformer energy hierarchy, which now includes also the CCSD(T) corrections. We observe that the CCSD(T) corrections are sensitive to the conformer geometry. They generally shift conformers down in energy toward the global minimum conformer. This reduces the energy spacing between the conformers. Conformers IIa and IIc are an exception. They remain at roughly the same relative energy to the global minimum, which is also of conformer type II. They subsequently trade places with other conformers in the hierarchy.

To validate our optimized conformer structures, we start with ref (30). The geometries reported in ref (30) were obtained at the MP2(FC)/aug-cc-pV(T^+^d)Z level, and we compare them against our PBE0 + MBD geometries. To standardize the comparison, we use the same conformer naming convention as in ref (47).

Among the top 10 most stable structures, ref (30) reports eight structures that we and ref (47) also found (see Table 2). These are IIb, IIa, Ib, I’b, Ia, III_β_b, III_β_c and III_α_b.^1^ The average differences of the dihedral angles between our and ref (30)’s geometries are Δ*d*_1_ = 4.6°, Δ*d*_2_ = 1.4°, Δ*d*_3_ = 2.8°, Δ*d*_4_ = 0.7° and Δ*d*_5_ = 3.0°. These small differences indicate that we indeed found the right conformers and that the PBE0 + MBD and MP2 geometries agree closely.

Reference (47) unfortunately, does not provide atomic coordinates for the reported conformers. To validate our optimized conformer structures against those of ref (47), we therefore performed MP4 single-point energy calculations with the same basis set 6–311++G(d,p) as in Ref (47), but for our PBE0 + MBD geometries. The results are reported in Figure 7b and Table 1.

**Table 1:**
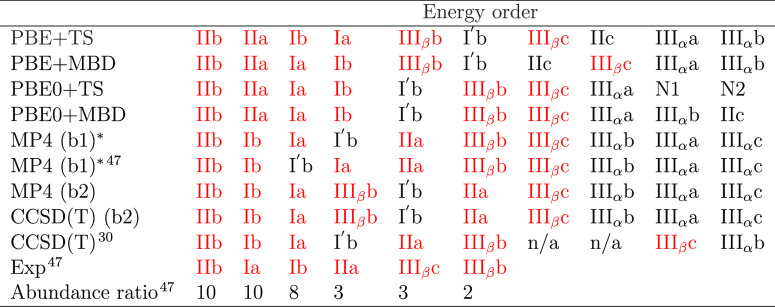
Energy Order of the 10 most Stable Conformers of Cysteine from our DFT, MP4, and CCSD(T) Computations, Ref (47) and Ref (30)^a^

a Our CCSD(T) and MP4 results are based on PBE0 + MBD structures. b1: 6-311++G(d,p) basis set, b2: aug-cc-pvtz basis set, *: vibrational energy corrections not included.

Figure 7b and Table 2 show that the energies of the two MP4 calculations (MP4(b1) and MP4(b1)^47^) agree within 4 meV for each conformer. This close match indicates that our conformer geometries agree very well with those of ref (47), validating our BOSS-based conformer search procedure.

**Table 2:**
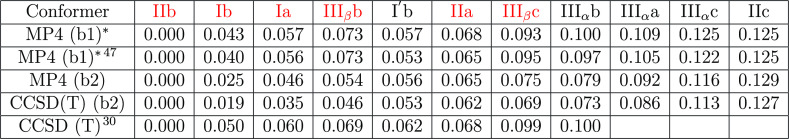
Predicted Low-Energy Conformers of Cysteine and Relative Energies with Respect to the Global Minimum in eV^a^

a b1: 6-311++G(d,p) basis set, b2: aug-cc-pvtz basis set,*: vibrational energy corrections not included.

Table 1 shows the final energy ranking of the top 10 most stable conformers in ref (47), ref (30) and our computational predictions. A more complete list of the low-energy conformers and their relative energy can be found in Table S1.

In our simulations, PBE + TS, PBE + MBD, PBE0 + TS, and PBE0 + MBD all found the correct global minimum structure IIb. PBE + TS, PBE0 + TS and PBE0 + MBD predicted the six experimental identified conformers among the top seven most stable structures, while PBE + MBD locates the six conformers among the top eight most stable ones.

**Figure 8:**
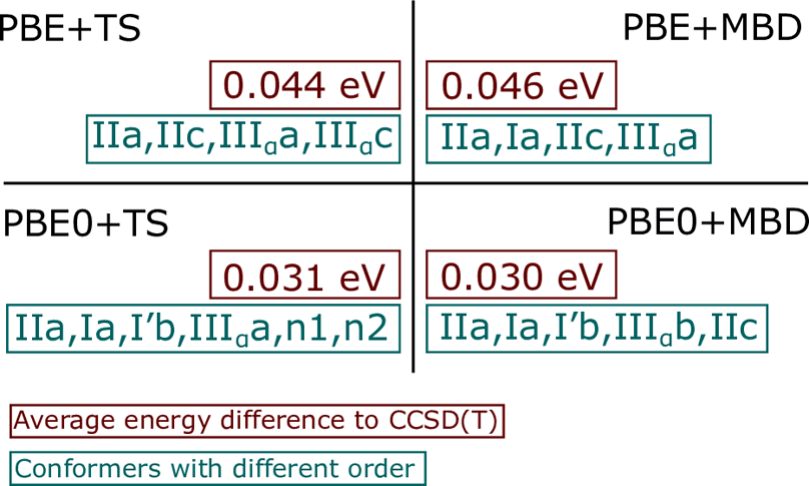
Summary of DFT results: each panel shows the average energy difference between the respective DFT functional and the CCSD(T) reference energies for the 10 lowest conformers. In addition, each panel lists the conformers that have a different order than in CCSD(T).

In Figure 8, we summarize the comparison across the four different exchange-correlation functionals we tested. Our reference are the CCSD(T) energies at the PBE0 + MBD geometries. In Figure 8, we list the conformers that have a different energy ordering in the DFT and CCSD(T). The energy differences between the cysteine conformers are extremely small. Therefore, it is no surprise that the DFT energy rankings differ from the CCSD(T) results. The accuracy of the different DFT functional are then evaluated by the energy differences comparing to CCSD(T), using the 10 lowest energy conformers in CCSD(T)). Comparing to CCSD(T), the average energy difference is 0.044 eV for PBE + TS, 0.046 eV for PBE + MBD, 0.031 eV for PBE0 + TS, and 0.030 eV for PBE0 + MBD (Figure 8). PBE0 is on average 0.01 eV more accurate than PBE. The difference between the different van der Waals treatments (TS or MBD) is an order of magnitude smaller (1 or 2 meV on average), but MBD is more than 10^2^ times more expensive than TS for cysteine. The influence of the different vdW treatments is negligible for a small molecule like cysteine; however, MBD may become important for accurate treatments of larger molecules, e.g., biomolecules. For cysteine, we can conclude that PBE + TS is sufficient for the conformer search.

Since BOSS is able to sample the configurational space very efficiently, we performed the whole conformer search at the PBE0 + MBD level. For larger molecules, it might become more economical to perform an initial BOSS-based conformer search at the PBE + TS level and to post-relax only a certain number of low-energy conformers with PBE0 + MBD.

Our CCSD(T) calculations produce a very similar energy ranking as the MP4 results in ref (47), as shown in Table 1. The only difference with ref (47) is the placement of I′b, III_β_b. If we use the same aug-cc-pvtz basis set, same geometries and same vibrational energy correction from our PBE0 + MBD simulations in both CCSD(T) and MP4, we get the same energy order. Therefore, the differences are not caused by the choice of CCSD(T) or MP4. Since we have validated that we have found very similar structures as ref (47) (Figure 7b), the difference may due to the fact that ref (47) did not include the entropy correction and used different basis sets.

Reference (30) reports two structures that are similar to IIa but do not appear in ref (47) or our conformer search. Except for these two new structures, the only difference between our CCSD(T) and the CCSD(T) results in ref (30) is the ordering of I′b and III_β_b. Again, the energy differences between the conformers in this range are extremely small, and ordering differences in our results and the reference can be ascribed to the slight difference of the conformer structures and computational settings. Reference^30^ used a different vibrational correction method and included the focal-point analysis to extrapolate the energies to the complete basis set limit.

Comparing our CCSD(T) results to the experiment, we note that the CCSD(T) ordering of IIb, Ib, and Ia as the three lowest energy conformers agrees with the experimental ordering derived from the relative abundance of the detected conformers. However, the order of Ia and Ib is switched, which is the same as the computational ranking in refs (47) and (30). For the next three conformers, the experiment finds IIa, III_β_b, and III_β_c, however, with much lower overall abundance than the first three conformers. The coupled cluster order is different with III_β_b, I′b, IIa, and III_β_c. These differences can be ascribed to the low experimental abundance, which might make an unambiguous classification difficult, or to additional experimental factors that are not taken into account in our simulations.

### Conformational Energy Hierarchy of Serine, Aspartic Acid, and Tryptophan

In this section, we applied our conformer search procedure with the PBE0 + MBD functional to serine, aspartic acid, and tryptophan. For comparison, we label their conformers in accordance with the corresponding reference.^63−65^

**Figure 9:**
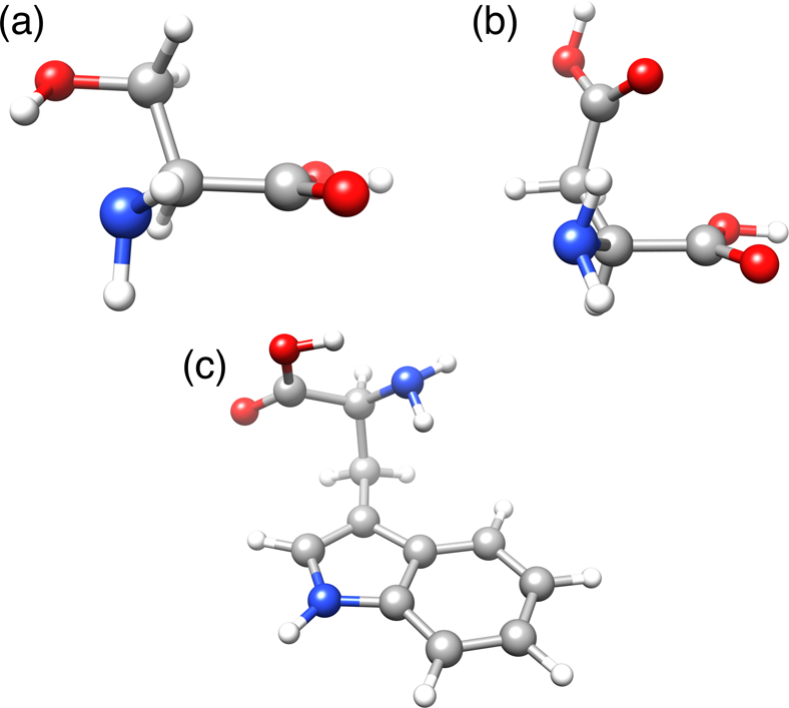
Global minimum structure of (a) serine, (b) aspartic acid, and (c) tryptophan.

The BOSS convergence of serine, aspartic acid, and tryptophan is similar to that of cysteine. Serine and aspartic acid converged in 1200 and tryptophan in 1000 iterations (Figure S8). We then followed the same procedure as for cysteine, i.e., we extracted and relaxed the local minima structures and included entropy corrections at 300 K. Finally, we added CC corrections to the 15 lowest energy conformers. The global minimum structures of the three molecules are shown in Figure 9, and the relative energy of the 10 lowest energy conformers are listed in Table 3.

**Table 3:**
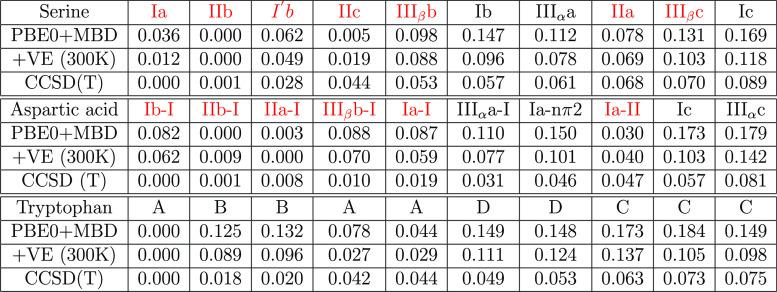
Predicted Low-Energy Conformers of Serine, Tryptophan, and Aspartic Acid and Relative Energies with Respect to the Global Minimum in eV^a^

a Aug-cc-pvtz basis set was used for the CCSD(T) calculations for serine and aspartic acid; 6-311++G(d,p) basis set was used for the CCSD(T) calculations for tryptophan.

For serine, we found the seven experimental detected conformers among the top nine most stable structures.^63^ The CCSD(T) energy ranking agrees well with the experimental population order, which is Ia > IIb > I′b > IIc > III_β_b ≈ III_β_c ≈ IIa.^63^ For aspartic acid, we found the six experimental reported conformers among the top eight most stable structures. Our order is close to the MP2-calculated conformer order in ref (64) (Table S3). Tryptophan has a more complicated structure and can form eight types of hydrogen bonds (A–H).^65^ Experiments and previous simulations have confirmed that the most stable one is A-type, which dominated the tryptophan population, followed by two B-type conformers.^65,66^ We also got the same order from our CCSD(T) energies.

To compare with reported computational results, we calculated the MP2 or MP4 energies of the three molecular conformers, using our PBE0 + MBD optimized structures and the same basis sets as in refs (63, 65). The relative energy and ranking of the 10 most stable molecular conformers are shown in Tables S2–S4.

For Serine, our MP4 results are very similar to ref (63). The only difference is that the order of IIc and I′b is switched. The average energy difference is 0.006 eV for all the conformers in Table S2.

For aspartic acid, the orders of IIb-I, III_β_c, Ia-nπ2, and Ib-III are different between our study and ref (64). However, the average energy difference is only 0.012 eV for all the conformers in Table S3, which reduces to 0.003 eV if we only consider the experimentally detected ones.

Reference (65) and we both found that the A and B types are more stable than other types for tryptophan. The average energy difference of A and B type conformers in Table S4 is 0.010 eV. These results proved that we had found similar conformer structures as the previous computational studies.

### Computational Efficiency

We close with a note on the efficiency of our new conformer search procedure without explicitly performing other search methods in this work. BOSS predicts a physically meaningful PES for the four amino acids with 5–6 degrees of freedom with only ∼1000 single-point DFT calculations. We can put this number of single-point calculations into perspective by considering that FHI-aims requires on average 30 geometry optimization steps to relax the structure of an organic molecule. The computational cost of 1000 single-point DFT calculations is therefore equivalent to approximately 30 DFT geometry optimizations.

From the PES, we extract all relevant low-energy conformers with the BOSS postprocessing minima search tool at a small computational expense. In this work, we consider approximately 80 local minima, each of which is geometry optimized with DFT. This amounts to 80 geometry optimizations, which is equivalent to approximately 2400 DFT single-point calculations.

Our total computational expense per DFT functional for a complete conformer search of cysteine is therefore 3400 DFT single-point calculations or equivalently about 100 geometry optimizations. Similar DFT steps were used to search the conformer of serine, aspartic acid, and tryptophan. This is a very small computational budget, compared to systematic^30^ or stochastic^32^ conformer search methods that need to relax thousands of structures. Supady *et al.* provided detailed numbers for a genetic algorithm (GA)-based conformer search of dipeptides.^32^ Their search encompasses between 4 and 6 degrees of freedom and is therefore similar to ours, as is the size of the molecules. The GA search requires between 20,000 and 60,000 single-point DFT calculations (referred to as force evaluations in ref (32)) depending on the size of the search space and the density of conformers in the energy hierarchy. Our BOSS-based procedure is a factor of 10 more efficient. A similar speed up was recently observed in a Gaussian-process-based structure search of oxidized graphene on the Ir(111).^67^ It is important to mention that different systems have different funneled PES, so the number of degrees of freedom is not the only important fact for conformer search. The comparison to the previous GA study^32^ is informative rather than quantitative.

## Conclusions

In summary, we propose a new conformer search procedure that combines the Bayesian optimization active learning with quantum chemistry methods. BOSS performs a global phase space search and finds all the relevant conformers in one run. Then, we refine the low-energy conformers by DFT structure relaxation, vibrational energy, and coupled cluster correction. We conclude that the DFT structure relaxation plays a major role in the refinement of the energy order. We also find that PBE0 gives slightly better results than PBE, but the difference between the TS and MBD van der Waals interactions are tiny for our system.

Unlike traditional conformer search methods, our approach is computationally tractable while retaining the accuracy of the chosen quantum chemical method throughout. This approach is most suitable for small molecules that require highly accurate and expensive quantum chemistry methods for conformer ranking. Extending the method to larger molecules with a much larger search space will require reliable dimension reduction strategy, either based on previous knowledge or computational techniques.

## Supporting Information Available

*xyz* coordinates of the low-energy conformers of cysteine, serine, aspartic acid, and tryptophan (ZIP)
(Table S1) Energy order of the low-energy conformers of cysteine; (Tables S2–S4) Predicted low-energy conformers and relative energies of serine (MP4), aspartic acid (MP2), tryptophan (MP2); (Figures S1 and S2) 2D-projected PES maps predicted by BOSS in the 5D case of cysteine; (Figure S3) total energy of cysteine conformers from BOSS, DFT single-point calculation, and after DFT optimization; (Figures S4–S6) Relative stability for all steps of the PBE + TS-based, PBE + MBD-based, and PBE0 + TS-based search; (Figure S7) DFT-calculated and transferred energy of the new structure each BOSS iteration predicted (aspartic acid); and (Figure S8) progression of the relative energy of predicted local-minima for a PBE0 + MBD BOSS run for serine, aspartic acid, and tryptophan (PDF)

## Footnotes

1 Using our classification standard, we classified the VI(n/a) conformer in Ref (30). to be IIb.
