# Supplementary material for: Efficient Amino Acid Conformer Search with Bayesian Optimization
Source: J Chem Theory Comput. 2021 Feb 12;17(3):1955–66. doi: 10.1021/acs.jctc.0c00648 (PMC8023666; doi:10.1021/acs.jctc.0c00648)
Supplement: Supplementary file 2 — ct0c00648_si_002.pdf [file ct0c00648_si_002.pdf]

Supplementary Material
Efficient Amino Acids Conformer Search with Bayesian Optimization

Lincan Fang, Esko Makkonen, Milica Todorović, Patrick Rinke,* and Xi Chen*

Department of applied Physics, Aalto University, Otakaari 1, FI-02150 Espoo, Finland

E-mail: patrick.rinke@aalto.fi; xi.6.chen@aalto.fi

Table of Contents

S1: The energy order of the low-energy conformers of cysteine

S2: The MP4-predicted low-energy conformers of serine, and relative energies.

S3: The MP2-predicted low-energy conformers of aspartic acid, and relative energies.

S4: The MP2-Predicted low-energy conformers of tryptophan, and relative energies.

Figure of Contents

S1: The 2-D (d1, d2) projected PES maps predicted by BOSS in the 5-D case of cysteine.

S2: The 2-D (d3, d4) projected PES maps predicted by BOSS in the 5-D case of cysteine.

S3: The total energy of cysteine conformers from BOSS, DFT single-point calculation,and after DFT optimization.

S4:Relative stability for all steps of the PBE+TS-based search.

S5: Relative stability for all steps of the PBE+MBD-based search.

S6: Relative stability for all steps of the PBE0+TS-based search.

S7:The DFT-calculated and the transferred energy of the new structure each BOSS iteration predicted (aspartic acid).

S8: Progression of the relative energy of predicted local-minima for a PBE0+MBD BOSS run for serine, aspartic acid and tryptophan.

Table S1: The energy order of the low energy conformers of cysteine

Different calculations											
Ref.1		CCSD(T)		PBE+TS		PBE+MBD		PBE0+TS		PBE0+MBD	
Type	E (eV)	Type	E (eV)	Type	E (eV)	Type	E (eV)	Type	E (eV)	Type	E (eV)
IIb	0.0	IIb	0.0	IIb	0.0	IIb	0.0	IIb	0.0	IIb	0.0
Ib	0.040	Ib	0.019	IIa	0.051	IIa	0.051	IIa	0.056	IIa	0.056
I′b	0.053	Ia	0.035	Ib	0.086	Ia	0.089	Ia	0.065	Ia	0.066
Ia	0.056	III_βb	0.046	Ia	0.087	Ib	0.089	Ib	0.067	Ib	0.067
IIa	0.065	I′b	0.053	III_βb	0.115	III_βb	0.117	I′b	0.091	I′b	0.092
III_βb	0.073	IIa	0.062	I′b	0.118	I′b	0.121	III_βb	0.094	III_βb	0.094
III_βc	0.095	III_βc	0.069	III_βc	0.126	IIc	0.124	III_βc	0.111	III_βc	0.116
III_αb	0.097	III_αb	0.073	IIc	0.126	III_βc	0.133	III_αa	0.120	III_αa	0.120
III_αa	0.105	III_αa	0.086	III_αa	0.135	III_αa	0.133	N1	0.132	III_αb	0.121
III_αc	0.122	III_αc	0.113	III_αb	0.140	III_αb	0.146	N2	0.143	IIc	0.128
IIc	0.125	IIc	0.127	N1	0.157	N1	0.156	N4	0.150	N1	0.130
				N4	0.165	N2	0.162	IIc	0.150	N2	0.137
				N2	0.167	N3	0.170	III_αc	0.158	N3	0.147
				III_αc	0.173	N4	0.173	N5	0.183	N4	0.158
				N5	0.203	III_αc	0.184	N6	0.188	III_αc	0.167

† The experimental detected conformers are marked by red.

Table S2: The MP4-predicted low-energy conformers of serine, and relative energies with respect to the global minimum in eV in our work and Ref. 1 (6-311++G(d,p) basis set, vibrational energies correction not included).

Serine	Ia	IIb	IIc	$I'b$	$III_{\beta}b$	$III_{\alpha}a$	IIa	Ib	$III_{\beta}c$	$III'_{\alpha}b$
MP4	0.000	0.001	0.019	0.024	0.042	0.063	0.073	0.097	0.093	
MP4[1]	0.000	0.003	0.017	0.037	0.059	0.061	0.073	0.086	0.094	0.096
Serine[1]	Ia	IIb	$I'b$	IIc	$III_{\beta}b$	$III_{\alpha}a$	IIa	Ib	$III_{\beta}c$	$III'_{\alpha}b$

† The experimental detected conformers are marked by red.

Table S3: The MP2-predicted low-energy conformers of aspartic acid, and relative energies with respect to the global minimum in eV in our work and Ref. 2 (6-311++G(d,p) basis set, 300K vibrational energies corrections included).

Aspartic acid	Ib-I	$III_{\beta}b$-I	IIb-I	IIa-I	Ia-I	Ia-II	Ia-nπ2	$III_{\alpha}a$-I	Ib-III	$III_{\beta}c$
MP2	0.000	0.017	0.023	0.032	0.033	0.039	0.055	0.061	0.078	0.095
MP2[2]	0.000	0.015	0.017	0.030	0.035	0.037	0.040	0.051	0.054	0.056
Aspartic acid[2]	Ib-I	IIb-I	$III_{\beta}b$-I	IIa-I	$III_{\beta}c$	Ia-I	Ia-nπ2	Ia-II	Ib-III	$III_{\alpha}a$-I

† The experimental detected conformers are marked by red.

Table S4: The MP2-predicted low-energy conformers of tryptophan, and relative energies with respect to the global minimum in eV in our work and Ref. 3 (6-311++G** basis set, 300K vibrational energies correction included).

Tryptophan	A	B	B	A	A	D	D	C	A	C
MP2 (b2)	0.000	0.034	0.035	0.050	0.061	0.063	0.067	0.086	0.093	0.095
MP2 (b2)[3]	0.000	0.024	0.025	0.039	0.051	0.059	0.060	0.064	0.065	0.069
Tryptophan[3]	A	B	B	A	D	E	A	C	A	A

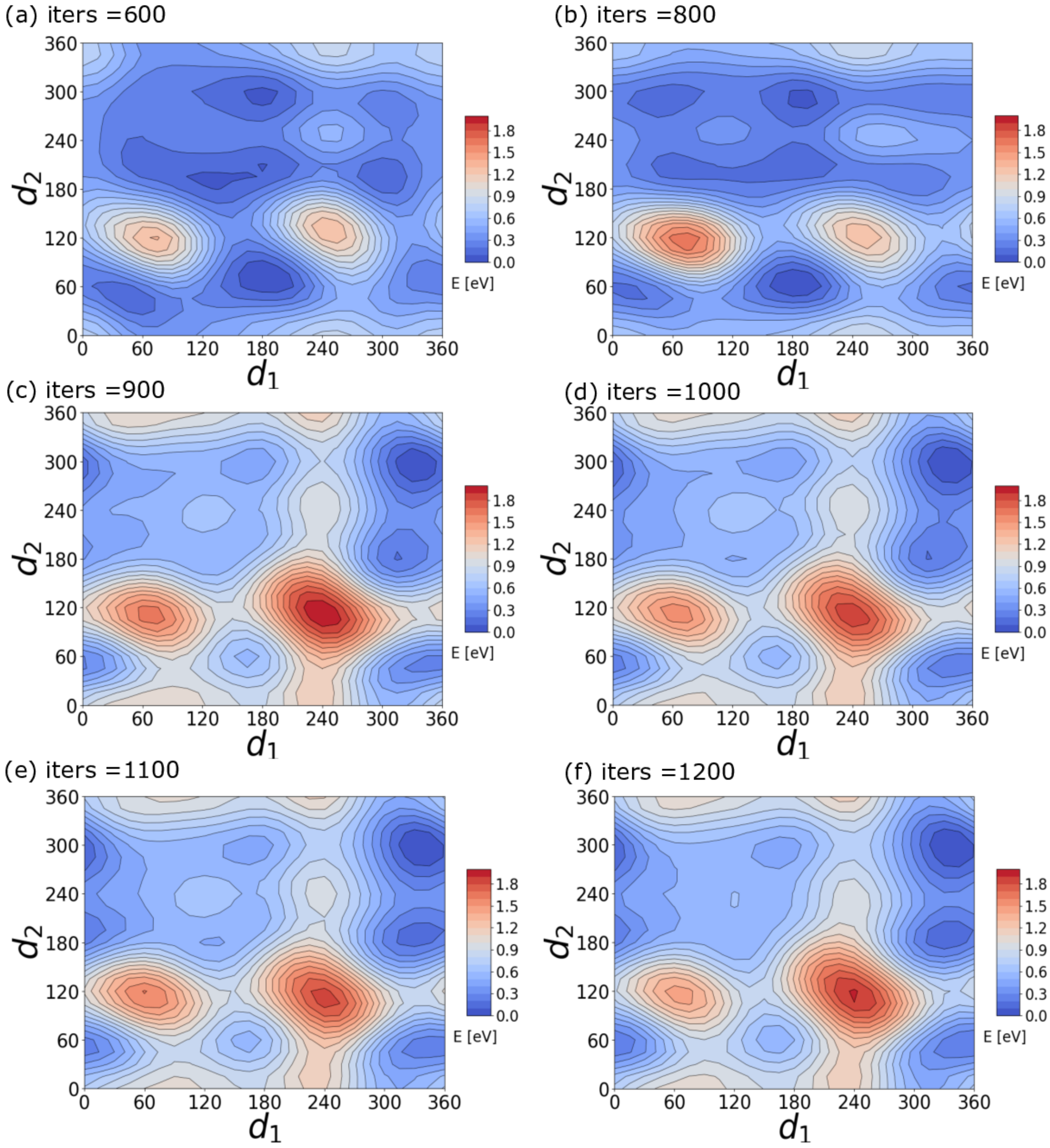

Figure S1: The 2-D (d1, d2) projected PES maps predicted by BOSS in 5-D case of cysteine from (a) 600 iterations, (b) 800 iterations, (c) 900 iterations, (d) 1000 iterations, (e) 1100 iterations, and (f) 1200 iterations. The PES maps become very similar after 900 iterations indicating the BOSS prediction of the whole PES has converged.

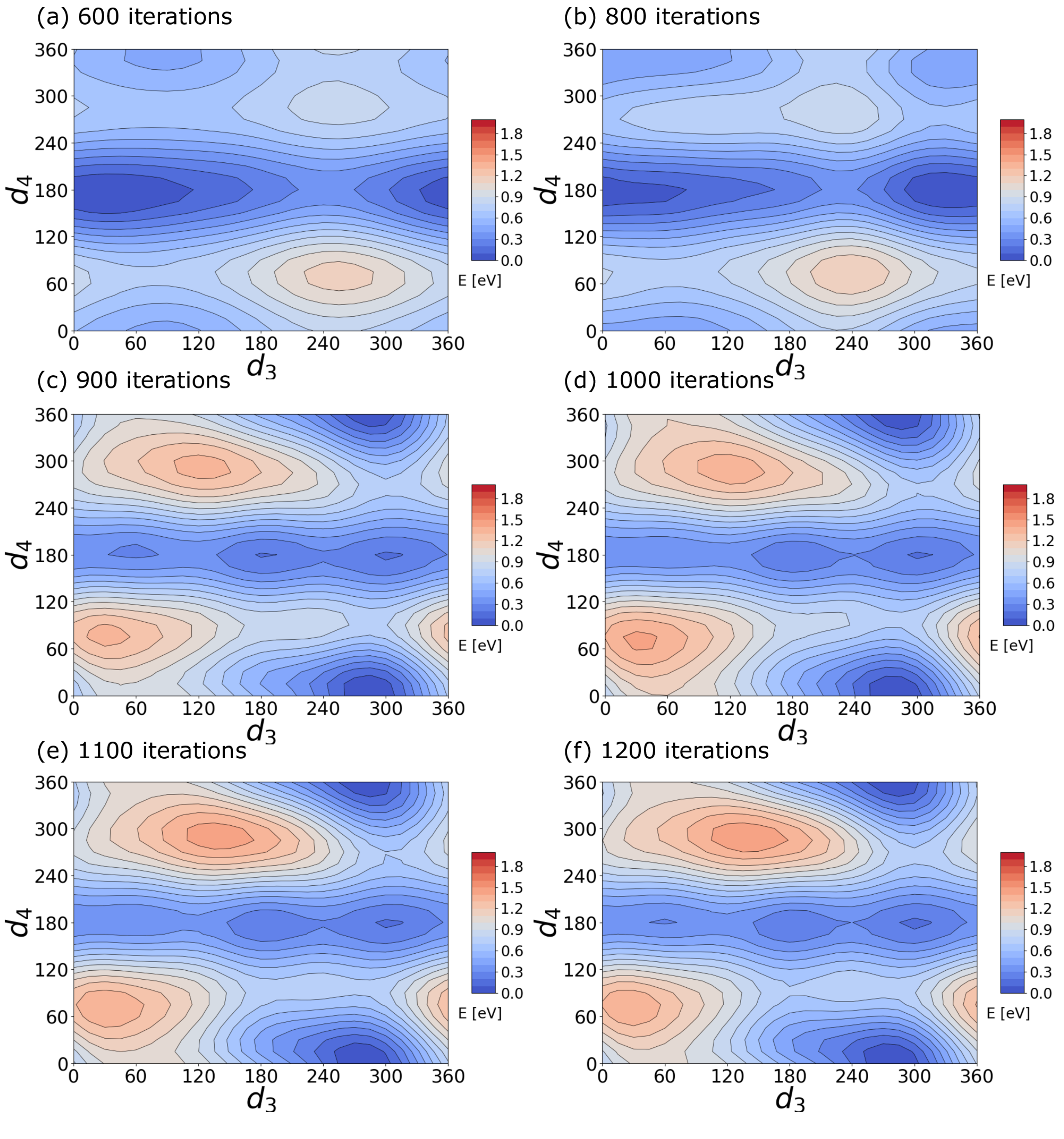

Figure S2: The 2-D (d3, d4) projected PES maps predicted by BOSS in 5-D case of cysteine from (a) 600 iterations, (b) 800 iterations, (c) 900 iterations, (d) 1000 iterations, (e) 1100 iterations, and (f) 1200 iterations. The PES maps become very similar after 900 iterations indicating the BOSS prediction of the whole PES has converged.

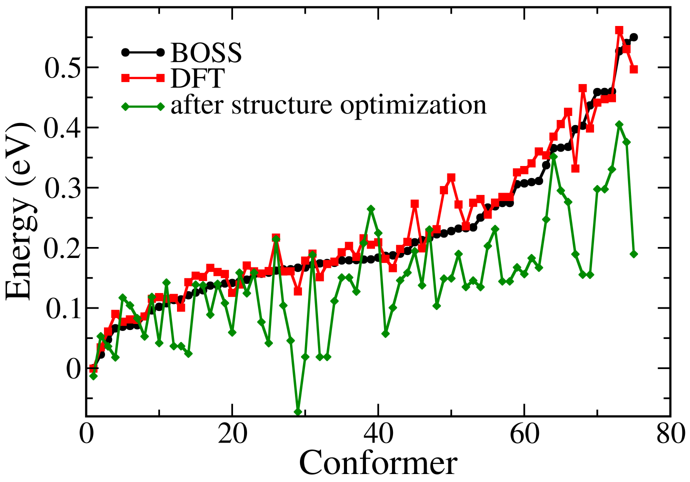

Figure S3: The total energy of cysteine conformers from BOSS, DFT single-point calculation, and after DFT optimization. The average energy difference between BOSS and DFT single-point calculation is 0.022 eV, and the average energe difference between the BOSS and DFT structure optimization is 0.084 eV for all the conforms in the figure. All the energy has been added the 19636.5607719 eV in the figure.

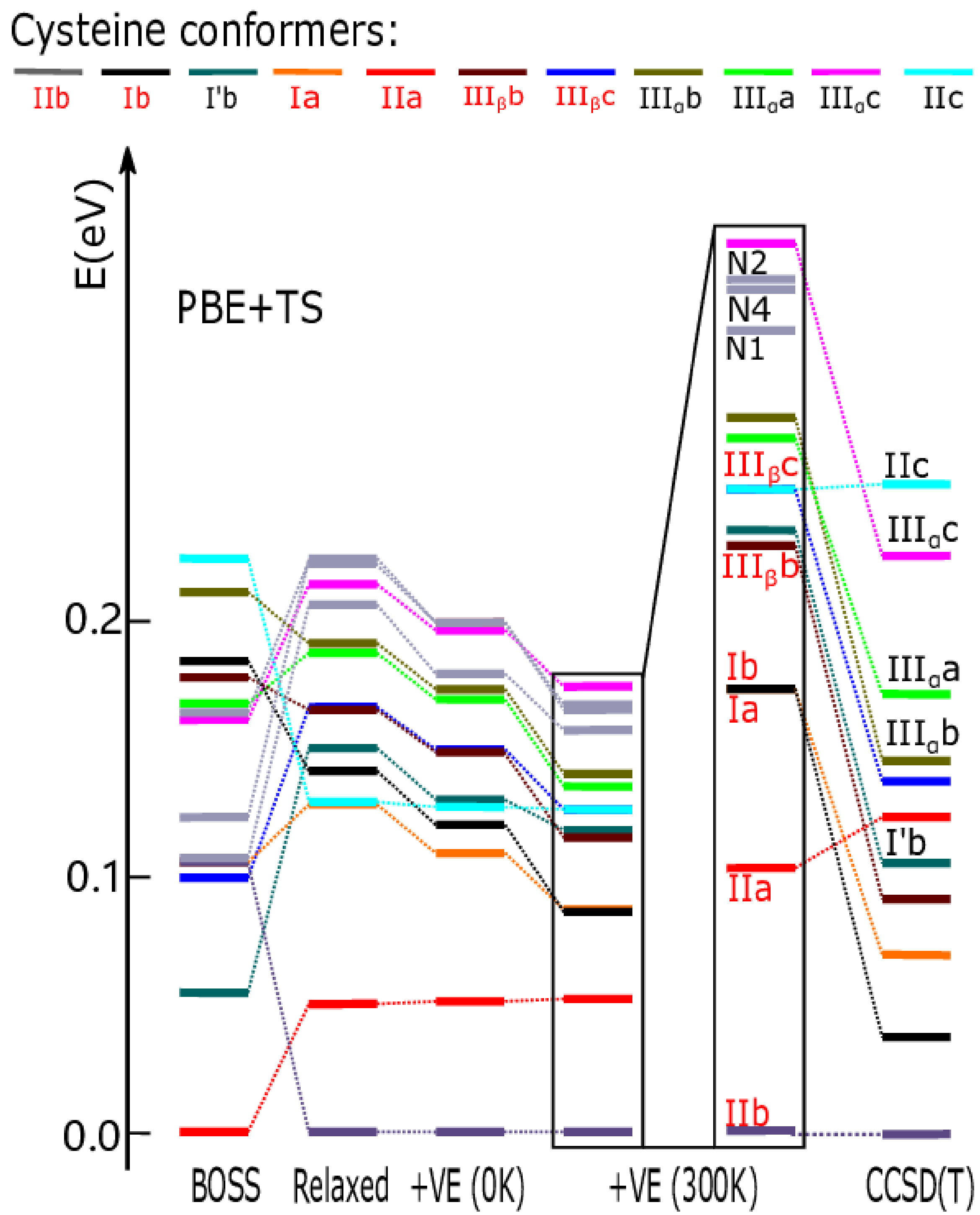

Figure S4: Relative stability for all steps of the PBE+TS-based search. From left to right: BOSS prediction, after structure optimization, after adding vibration energy at 0 K (+VE (0K)) and after adding vibration energy at 300 K (+VE (300K)). The two most right ones are +VE (300K) and the energy order of CCSD(T) result but enlarged 2 times. For each step, the energy of the most stable structure defines the zero of the energy.

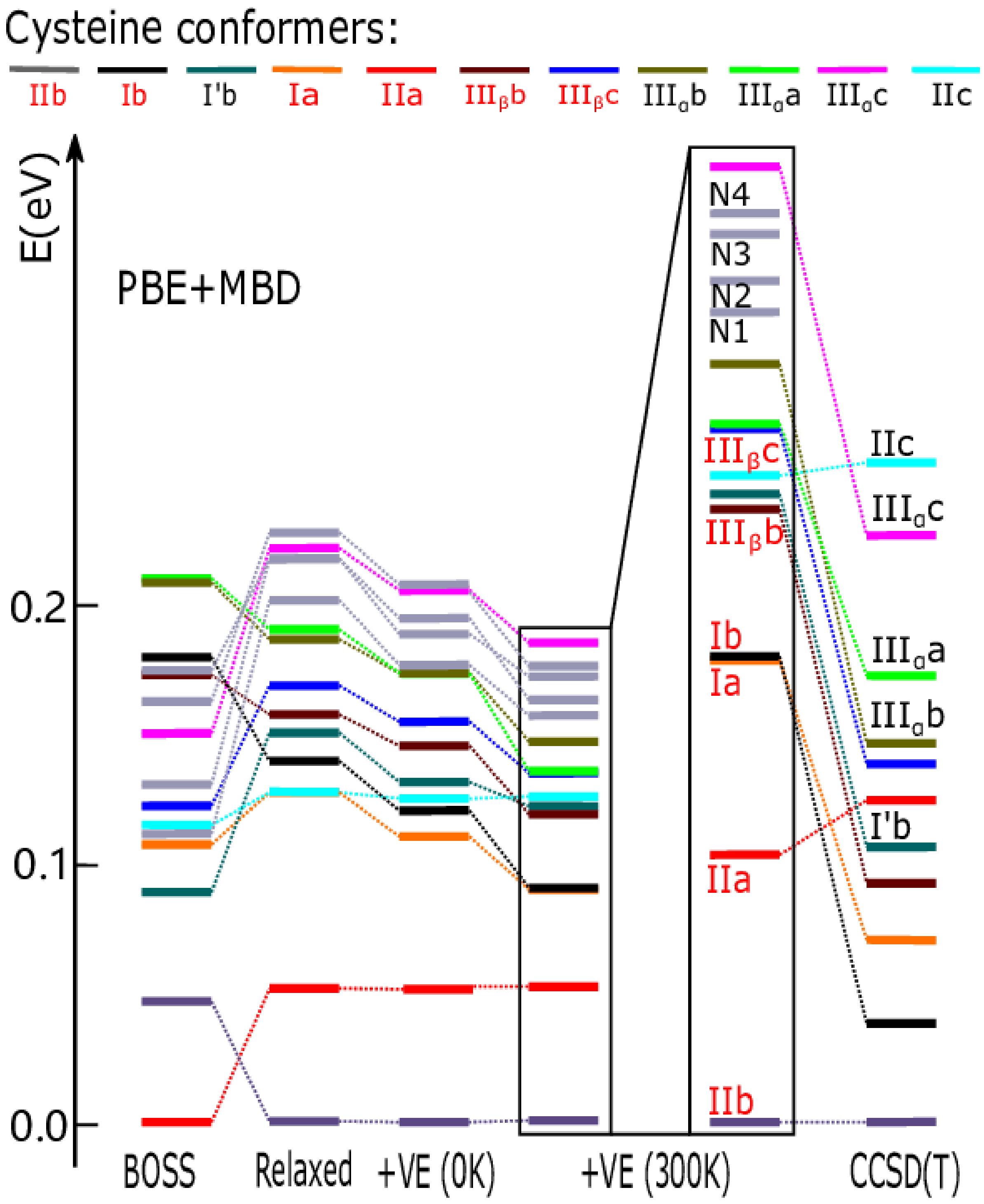

Figure S5: Relative stability for all steps of the PBE+MBD-based search. From left to right: BOSS prediction, after structure optimization, after adding vibration energy at 0 K (+VE (0K)) and after adding vibration energy at 300 K (+VE (300K)). The two most right ones are +VE (300K) and the energy order of CCSD(T) result but enlarged 2 times. For each step, the energy of the most stable structure defines the zero of the energy.

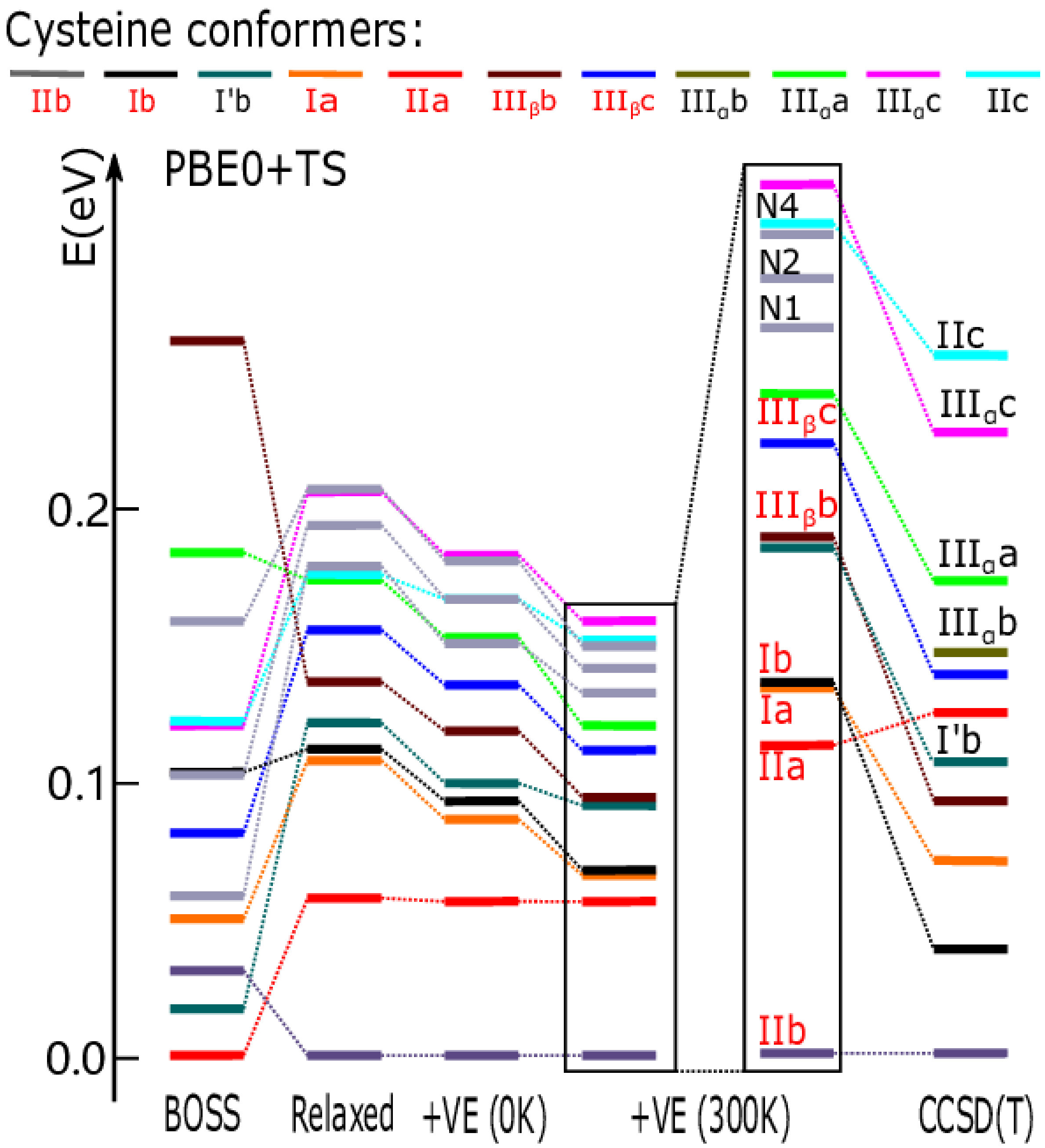

Figure S6: Relative stability for all steps of the PBE0+TS-based search. From left to right: BOSS prediction, after structure optimization, after adding vibration energy at 0 K (+VE (0K)) and after adding vibration energy at 300 K (+VE (300K)). The two most right ones are +VE (300K) and the energy order of CCSD(T) result but enlarged 2 times. For each step, the energy of the most stable structure defines the zero of the energy.

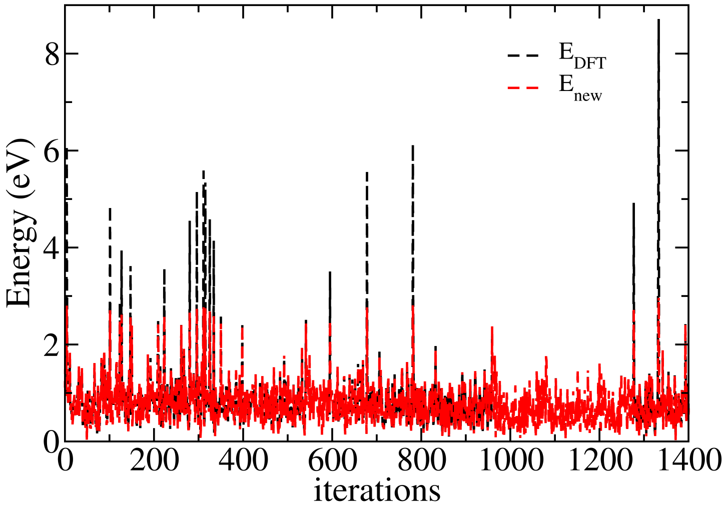

Figure S7: The DFT-calculated and the transferred energy of the new structure each BOSS iteration predicted (aspartic acid). The total energy of the structure calculated in iteration 0 was set to be 0 eV.

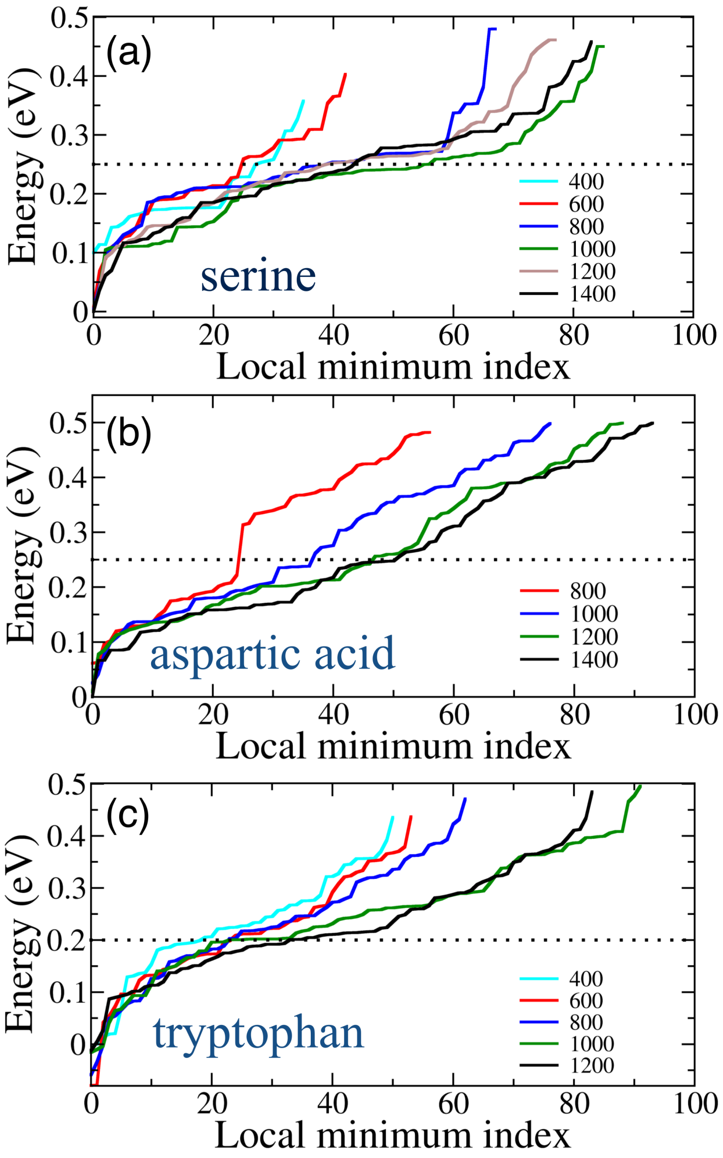

Figure S8: Progression of the relative energy of predicted local-minima for a PBE0+MBD BOSS run. Shown are intermediate curves for (a) serine, (b) aspartic acid and (c) tryptophan.

References

(1) Blanco, S.; Sanz, M. E.; López, J. C.; Alonso, J. L. *Proceedings of the National Academy of Sciences* **2007**, *104*, 20183–20188.

(2) Sanz, M. E.; Lpez, J. C.; Alonso, J. L. *Phys. Chem. Chem. Phys.* **2010**, *12*, 3573–3578.

(3) Huang, Z.; Lin, Z. *The Journal of Physical Chemistry A* **2005**, *109*, 2656–2659.
